# Implantation of a Bi-Ventricular Pacing System in the Setting of Dextrocardia with Situs Inversus Totalis

**Published:** 2010-01-07

**Authors:** Amit A Doshi, Stephen C Cook, John D Hummel

**Affiliations:** The Ohio State University, Columbus, OH, USA

**Keywords:** Dextrocardia, Bi-Ventricular pacing, Persistent superior vena cava

## Abstract

Dextrocardia with situs inversus totalis is a rare disorder but is frequently associated with anomalous venous return.  Pacemaker/Internal Cardioverter Defibrillator implantation in this population can be difficult given the difficult venous anatomy.  This case illustrates how beforehand knowledge of the venous anatomy by cardiac MRI can facilitate device implantation.

## Introduction

Dextrocardia with situs inversus totalis is a condition with  reverse malposition of the visceral organs with a prevalence of 1 to 2 per 10,000 people in the United States [1]. Less than 10% of these patients have associated congenital cardiac defects, but of those that do occur, anomalous venous return is common.

Venous anomalies can arise in the form of abnormal pulmonary venous return or a persistent vena cava. In the general population, a left persistent superior vena cava (PSVC) is a congenital anomaly present in approximately 0.5% of the general population [2]. In nearly 60% of these cases, there is an innominate vein which bridges the two superior vena cava [3]. Presence of a PSVC can make transvenous lead implantation difficult, however when present with favorable coronary sinus anatomy it may facilitate lead positioning [4]. We report a case of a bi-ventricular implantable cardioverter defibrillator (CRT-D) implantation in a patient with dextrocardia and situs inversus totalis as well as a persistent right SVC.

## Case Report

The patient was a 38 year old caucasian man with known dextrocardia and situs inversus totalis who initally presented with increasing dyspnea following a febrile illness. Initial evaluation including cardiac catheterization revealed the patient to be suffering from a severe non-ischemic, dilated cardiomyopathy with an estimated ejection fraction of 15%. Despite optimization of his heart failure regimen the patient continued to demonstrate progressive congestive heart failure, while cardiac MRI six months after the initial diagnosis revealed persistent left ventricular dysfunction with an estimated ejection fraction of 17% and 12-lead electrocardiogram showed a LBBB with QRS duration of 154 msec. The patient was subsequently referred for implantation of a CRT-D.

**Figure 1:**
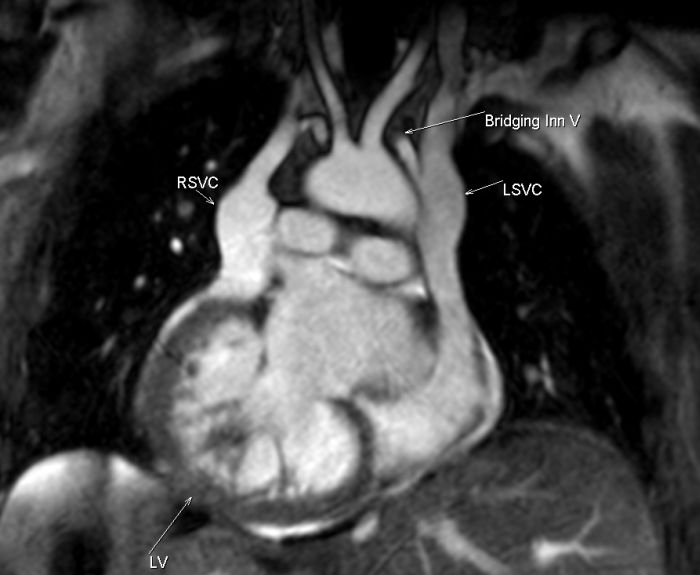
Coronal Cardiac MRI image of the patient showing the presence of the right persistent superior vena, left superior vena cava and bridging inominate vein.

Given the dextrocardia, a right sided approach was undertaken to optimize defibrillation vectors. Triple cannulation of the right axillary vein was performed. Cardiac MRI had revealed the presence of a right persistent superior vena cava (RPSVC) and innominate vein bridging to the proper (given the diagnosis of situs inversus totalis) left superior vena cava (LSVC) (Figure 1).

**Figure 2:**
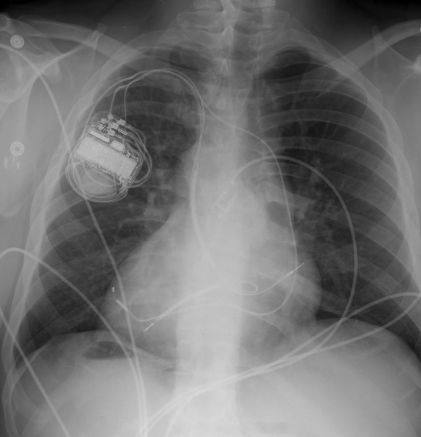
PA projection CXR showing dextrocardia with situs inversus totalis and final placement of the CRT system

An initial attempt was made to introduce an active fixation dual coil ventricular defibrillation lead (Endotak Reliance Goretex™, model 0184, Boston Scientific Corp., St. Paul, MN) and an active fixation bipolar atrial lead (Medtronic Capsurefix Novus 5076™, Medtronic Corp.) via the innominate vein draining into the left superior vena cava. This approach failed as there was inadequate innominate vessel caliber to allow free movement of the leads. Subsequent venography showed that the right PSVC drained into the right atrium via the coronary sinus. (Figure 1 and Figure 2). The dual coil lead defibrillator lead was redirected via the right PSVC and was placed in the RV apex. Capture threshold measured 0.6 V at 0.5ms with an impedence of 675Ω, sensing amplitude was 30.0mV with a slew rate of 4.0 V/s and high-voltage shock impedance was 44Ω.

The bipolar atrial lead was also introduced via the right PSVC into the right atrium. The capture threshold measured 0.7 V at 0.5ms with an impedence of 655Ω, sensing amplitude was 1.9mV with a slew rate of 0.5 V/s.

The LV lead (Medtronic 4194™, Medtronic Corp., St. Paul, MN) was also introduced via the RPSVC, however it was not feasible to place this lead into the lateral branch off of the coronary sinus given the acute angulation of the side branches off the main body of the coronary sinus when approaching from the RPSVC. Subsequently, the coronary sinus was approached via the innominate vein with a pair of telesoping sheaths and the LV lead was easily placed in a posterior lateral branch off the main body of the coronary sinus (Figure 2). The capture threshold measured 0.8 V at 0.5ms with an impedence of 1057Ω, sensing amplitude was 30.0mV with a slew rate of 4.0 V/s. The leads were connected to a CRT-D device (Medtronic C154DWK Concerto™, Medtronic Corp.), in the right pectoral area. Defibrillation threshold testing was performed with induction by burst pacing and was found to be ≤ 12J.

## Discussion

**Figure 3:**
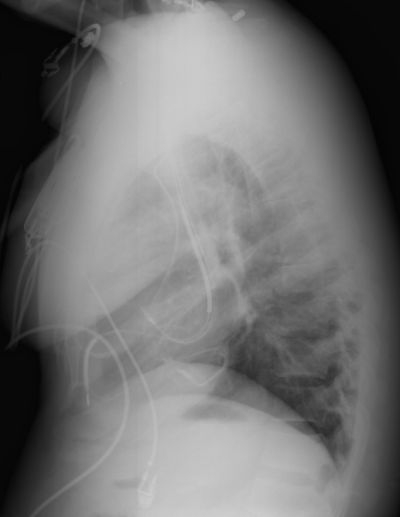
Lateral projection CXR showing final placement of the CRT system with separation of the RV and LV leads

Dextrocardia with situs inversus totalis is a rare condition, however the presence of a persistent contralateral SVC provides the opportunity for important learning points for implantaion of CRT-D devices in patients with congenital heart disease. Historically, the presence of a left PSVC has made tranvsvenous lead implantation very difficult [2].  In this patient, the acute angle of entrance from the superior aspect of the coronary sinus into its side branches presented a significant challenge for LV lead placement. However, it is important to note that many patients with a PSVC will have bridging to contralateral superior vena cava via an innominate vein. In this case, initial attempts were made to place the RV and RA lead via the innominate vein, however due to the small vessel caliber this approach was abandoned. With our knowledge that this patient had a RPSVC we were able to succesfully place both the RA and RV lead via this approach. The presence of the innominate vein in this case facilitated placement of the LV lead via the coronary sinus and into a posterior lateral branch without the challenge of hyperacute angulation of the side branch.  We debated whether to place a dual or single coil defibrillator lead given the thin coronary sinus wall, but given the coating of the coils with Goretex™ to decrease the risk of extraction and the potential advantage in defibrillation energy requirements we chose to place the dual coil lead.  What was initially challenging anatomy, actually facilitated placement of the LV lead as there were no competing leads for the innominate vein use by the LV lead (Figure 3). In conclusion, presence of a PSVC and bridging innominate vein may facilitate implantation of CRT-D devices in complex congenital heart disease.
